# Tailored surgical approaches for spinal chordomas: A multidisciplinary perspective

**DOI:** 10.1007/s00701-024-06290-w

**Published:** 2024-10-03

**Authors:** Lukas Klein, Pavlina Lenga, Philip Dao Trong, Helena Kleineidam, Sandro M. Krieg, Basem Ishak

**Affiliations:** 1 Department of Neurosurgery, Heidelberg University Hospital, Heidelberg, Germany; 2 Medical Faculty of Heidelberg, Heidelberg University Hospital, Heidelberg, Germany; 3 Department of Neurosurgery, University of Heidelberg, Im Neuenheimer Feld 400, 69120 Heidelberg, Germany

**Keywords:** Spinal chordoma, Tailored approached, Surgery, Complications, Morbidity

## Abstract

**Introduction:**

The treatment of spinal chordomas presents a significant challenge due to their resistance to both radiotherapy and chemotherapy as well as the complexity of the surgical procedures required. This study presents a series of cases of primary spinal chordomas, focusing on the development of a personalized therapeutic strategy that is tailored to each patient's unique clinical status. This approach aims to ensure that treatments are optimally aligned with the patient’s overall prognosis and surgical eligibility.

**Methods:**

This retrospective study analyzed 14 patients with primary spinal chordomas treated at our institution. We evaluated surgical strategies, clinical outcomes, and survival rates, The therapeutic strategy was formulated after interdisciplinary conferences with sarcoma management specialists. Data were collected on patient demographics, surgical details, postoperative outcomes, and follow-up status.

**Results:**

All patients presented with neurological deficits preoperatively, which generally improved post-surgery. The study included a detailed analysis of two distinct surgical approaches: five patients underwent en bloc resection with dorsal stabilization and nine received decompression only. Patients undergoing en bloc resection showed a reduced need for additional surgery due to the comprehensive removal of the tumor. As anticipated, 40% of the patients who underwent decompression experienced tumor progression within the first three months. However, given the poor overall prognosis, the objective of maintaining neurological function was achieved.

**Conclusions:**

Surgical en bloc resection offers a viable and effective intervention for spinal chordomas, enhancing neurological function. It is imperative to tailor treatment strategies to individual prognoses, integrating insights from multidisciplinary discussions that meticulously evaluate surgical risks. This collaborative approach aids in selecting the most appropriate surgical technique tailored to each patient’s specific condition.

## Introduction

Chordomas are rare and indolently progressive mesenchymal tumors, accounting for 1–4% of all bone malignancies. It originates from embryonic notochord remnants and is exclusively located within the axial skeleton [23]. The overall incidence is 0.08 per 100,000 individuals, exhibiting a male predominance and a peak occurrence between the ages of 50 and 60 years[17, 23]. The anatomical distribution is primarily in the sacrum (50–60%), followed by the spheno-occipital region (25–30%), the cervical spine (10%), and the thoracolumbar vertebrae (5%)[16, 26]. It is noteworthy that the tumor's gradual increase in volume often results in a delayed presentation of neurological deficits, which consequently may lead to a postponed diagnosis. However, once neurological deficits are present, an expeditious commencement of treatment is crucial.

Chordomas are notoriously resistant to radiotherapy and chemotherapy, making surgical resection the mainstay of treatment [8, 23]. Current evidence suggests that en bloc resection with wide margins is the superior surgical approach to achieve disease-free survival in patients with chordoma of the mobile spine or sacrum [5, 11]. Nevertheless, the distinctive anatomical and functional characteristics of the spine pose a formidable challenge in achieving wide or marginal resection, even for very experienced surgeons, resulting in severe complications or even mortality [5, 8, 18, 19]. This poses the question of how to optimally treat patients, balancing the need for maximal resection to preserve patient's quality of life and prolong survival rates. In case of residual disease post-surgery, adjunctive radiotherapy is advisable. However, in the absence of established protocols or guidelines, treatment strategies are predominantly based on empirical evidence and small retrospective series. It is posited that early adjunctive radiotherapy may be pivotal in gaining early control of the disease [16].

Given the scarcity of comprehensive literature on treatment strategies for spinal chordomas, our study intents to present a case series of primary spinal chordoma patients with an emphasis on developing personalized therapeutic strategies tailored to each patient’s clinical status. This targeted approach is designed to ensure that treatments are optimally aligned with patients' overall prognosis and surgical eligibility, thereby aiming to deliver the most effective therapy possible.

## Methods

### Study design and inclusion criteria

This is a retrospective study collecting data on spinal chordomas from our institutional database (approval number S518/2023) and adhered to the principles of the Declaration of Helsinki. Patients aged 18 years or older with primary spinal chordoma were included in the study. Patients under 18 years of age, patients with coexisting intracranial or cervical disease, secondary spinal tumors, or incomplete data were excluded.

The study included a thorough review of patient demographics, comorbidities, American Society of Anesthesiologists (ASA) scores, duration of surgery and number of spinal levels treated. Length of hospital stay, time in the intensive care unit, cases of readmission, need for reoperation and mortality rates were recorded. The Karnofsky Performance Index (KPI) was used to assess changes in a patient's condition by assigning scores for their ability to perform specific tasks according to the following: 100, normal condition, no discomfort; 70, inability to perform activities of daily living; 50, requires substantial assistance; 40, disabled; 30, hospitalization required; and 0, deceased ka[13]r. The extent of spinal cord injury was assessed using Frankel grades (FG): A, complete motor and sensory loss below the level of spinal cord dysfunction; B, preserved sensation but no motor activity; C, preserved but non-functional motor function; D, useful preservation of motor function; and E, normal motor function [10]. Routine clinical and radiological assessments, including MRI scans of the spinal cord, were performed before discharge and at three months postoperatively and yearly thereafter to monitor for tumor recurrence.

### Decision making

Various factors such as surgeon preference, tumor location, size and aggressiveness were considered in the choice of surgical procedure. Oncologic considerations included the comparison of surgical options with radiotherapy and chemotherapy, the feasibility of complete resection after neoadjuvant therapy and the response to steroids. The surgical approach (en bloc resection or laminectomy with tumor removal) was planned in interdisciplinary conferences, taking into account the overall prognosis of the disease. It should be emphasized that patients who underwent surgical decompression only were in a palliative stage with a poor prognosis, as determined by the internal tumor board. Therefore, the primary objective of the surgery was to maintain neurological function and prevent any further neurological decline. Adjuvant chemotherapy and radiotherapy were administered as salvage therapies, predominantly in patients after incomplete resection or in the palliative phase at the discretion of the treating physician.

#### Tumor extent and surgical approach

##### Sector involvement

Tumors were evaluated for their invasion into adjacent vertebral sectors. This classification was crucial for determining the extent of surgical resection needed. For instance: Patients with tumors affecting only the vertebral body underwent laminectomies.

More extensive involvement, including multiple vertebrae or critical areas like pedicles, necessitated complex reconstructions such as vertebrectomy combined with dorsal instrumentation [6].

##### Surgical margins

Aimed for wide margins where feasible, with some cases requiring more conservative margins due to tumor location and patient condition.

##### Spinal stability and reconstruction:

- For Partial Resections: We typically used vertebral body replacement systems or bone grafts to fill the void left by the resected tumor, supported by pedicle screw fixation to provide immediate structural stability.
- For Total Resections: More extensive reconstruction was required, often involving custom-fabricated implants or expandable cages to replace larger segments of the vertebral column, supplemented by long-segment fixation using rods and screws that span multiple vertebral levels above and below the site of resection.

##### Post-surgical stability

All cases involved assessments of spinal stability, with interventions tailored to the extent of tumor resection and the specific vertebrae involved. Reconstruction techniques varied from simple rod and screw assemblies to more complex systems involving cages and grafts, depending on the extent of resection and the specific needs for spinal integrity.

Outcomes and Follow-up: Postoperative monitoring focused on symptom management, functional recovery, and surveillance for potential recurrence. The effectiveness of the surgical approach was evaluated based on pain relief, functional status, and radiologic assessments of spinal alignment and integrity.

### Statistical analysis

Categorical variables are presented as numbers and percentages. Continuous variables are presented as means ± standard deviations, and the Shapiro–Wilk test was used to test whether their distribution was normal. Baseline characteristics, duration of surgery, number of spinal segments treated, perioperative and postoperative complications, length of stay (LOS), ICU stay, readmissions, reoperations and mortality were reported.

## Results

**Fig. 1:**
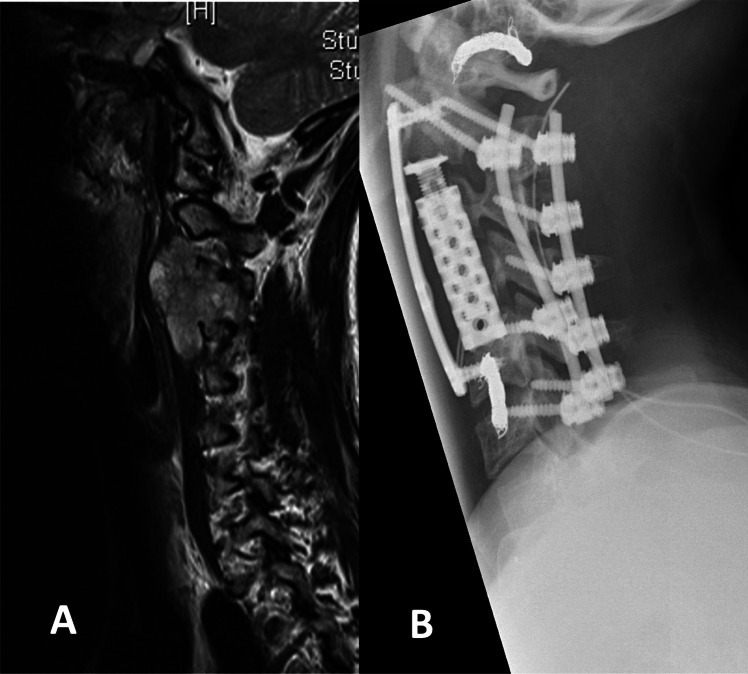
**A** Sagittal T2-weighted MRI: This image displays a hyperintense tumor mass extending from the C3 vertebra, illustrating the extent and nature of the spinal chordoma before surgical intervention. **B** Postoperative X-rays: These images show dorsal instrumentation extending from C2 to C6 and a vertebrectomy performed from the ventral aspect with a plate

This study included 14 patients suffering from primary spinal chordomas over a period of 17 years. Mean age was 56.2 ± 16.0 years, showing a slight male predominance (n = 8/14, 57.1%). Of these patients, all patients presented with back pain, three patients (21.4%) with a Frankel grade A and one (7.1%) with a Frankel grade B. The tumors were predominantly located in the cervical spine (41.2%), followed by sacral region (28.6%), thoracic spine (14.3%) and thoracolumbar spine (7.1%). A detailed overview of patient characteristics is presented in Table 1. Figure 1 presents a case undergoing dorsoventral instrumentation of the cervical spine.

**Table 1:** Baseline characteristics

Characteristic	Value
Number of patients	14
Age, months (mean, SD,range)	56.2 (16.0;24–62)
Sex (n, %)	
Male	8 (57.1)
Female	6 (43.9)
Body mass index, kg/m^2^ (mean, SD)	28 (3.7)
ASA class (mean, SD)	2.4 (0.8)
Symptoms	
Frankel Grade	
A	3 (21.4)
B	1 (7.1)
C	0 (0.0)
D	8 (57.1)
E	2 (14.3)
KPI (mean, SD)	61 (21.0)
Level of Spinal Cord Compression (n, %)	
Cervical	7 (41.2)
Thoracic	2 (14.3)
Thoracolumbar	1 (7.1)
Sacral	4 (28.6)

^*^ ASA, American Society of Anesthesiologists; KPI, Karnofsky Performance Index; MS, motor score of the American Spinal Injury Association grading system; SD, standard deviation

## Surgical characteristics and clinical assessments

Mean operative time was 223 min, with a range of 100 to 445 min with a standard deviation (SD) of 94 min. Nine Patients (64.2%) underwent surgical decompression, while 5 patients (35.7%) received a decompression with ventrodorsal instrumentation and en bloc resection. Mean number of spinal segments treated was 2.5 ± 1.6. Mean ICU stay was less than two days, and total hospital stay averaged 14.0 ± 9.6 days. Postoperative neurological and functional evaluations, as represented by the FG and KPI, indicated notable improvements with detailed data presented in Table 2 and Table 3. The average follow-up period was 56.3 months, with a standard deviation of 60 months. No patient required further surgical interventions for secondary instability. The detailed surgical management for patients without recurrence surgery is presented in Table 4.

**Table 2:** Peri- and postoperative surgical characteristics

Characteristic	All (n = 14)
Surgical duration, minutes (mean, SD)	223 (94.0)
Surgical approach (n, %)	
Decompression	9 (64.3)
Decompression + ventrodorsal instrumentation (en bloc resection)	5 (35.7)
Number of levels decompressed(n, %)	2.5 (1.6)
Hospital stay, days (n, %)	14 (9.6)
ICU stay, days (n, %)	0.6 (0.9)
Mortality	
In-hospital (n, %)	1 (7.1)
90-day (n, %)	1 (7.1)
30-day readmission (n, %)	1 (7.1)
Postoperative Frankel Grade (n, %)	
A	0 (0.0)
B	4 (28.6)
C	0 (0.0)
D	6 (42.9)
E	4 (28.6)
Postoperative KPI (mean, SD)	68 (18)
Chemotherapy after surgery (n, %)	1 (7.1)
Radiation after surgery (n, %)	9 (64.3)

The values are indicated as mean (SD) unless otherwise indicated

ICU, intensive care unit; MS, motor score of the American Spinal Injury Association grading system

^*^Comparison between intra-and extradural pathologies

**Table 3:** Occurrence of adverse events

Event	All (n = 14)
Pneumonia	1 (7.1)
Ischemic infarction	1 (7.1)
Revision surgery (local recurrence)	6 (42.9)

**Table 4:** Detailed Surgical Management and Outcomes for Patients with Spinal Chordomas Without Further Surgery

Patient Age	Tumor Location	Extent of Tumor Involvement	Surgical Intervention	Surgical Margins Achieved	Stability Measures Implemented	Postoperative Outcomes
54 years	Cervical (C0-C5)	Extensive involvement across multiple vertebrae	Dorsal instrumentation	Aimed for wide margins where possible	Rods and screws for long-term stabilization	Pain managed, improved spinal stability
53 years	Sacral region	Localized tumor presence	Laminectomy	N/A (palliative intent)	None due to localized approach	Effective pain relief, maintained functional mobility
54 years	Sacral region	Localized tumor presence	Laminectomy	N/A (palliative intent)	None due to localized approach	Effective pain relief, maintained functional mobility
52 years	Cervical	Localized involvement	Laminectomy	N/A (palliative intent)	None due to localized approach	Symptom management, pain relief
56 years	Sacral region	Localized tumor presence	Laminectomy	N/A (palliative intent)	None due to localized approach	Effective pain relief, maintained functional mobility
61 years	Thoraco-lumbar (T11-L3)	Involvement across multiple levels	Dorsal instrumentation	Aimed for wide margins where possible	Extensive instrumentation for spinal integrity	Enhanced structural support, improved mobility
30 years	Thoracic (Th11-Th12)	Localized to two levels	Laminectomy	N/A (palliative intent)	None required for localized surgery	Decompression achieved, significant pain relief
56 years	Lumbo-sacral (L2-S2, L4)	Extensive multi-level involvement	Dorsal instrumentation and vertebrectomy at L4	Wide margins aimed to remove all affected tissue	Complex reconstruction with cages and grafts	Marked pain relief, restored mobility and stability

Furthermore, we analyzed the histological findings, revealing that the majority of patients were diagnosed with conventional chordoma (Table 5).

**Table 5:** Histological Profiles and Proliferative Indices of Chordoma Variants

ID	Histology Type	Localization	Surgical Approach	Recurrence surgery	S100	AE1/3	EMA	MIB-1	Ki-67 (%)	Brachyury	Chemotherapy	Radiation
1 (76y)	Conventional	Cervical	Laminectomy	yes	+	+	+	Isolated cells marked	< 5%	+	No	Yes
2 (53y)	Conventional	Sacral	Laminectomy	no	+	+	+	< 5%	N/A	+	No	Yes
3 (54y)	Conventional	Sacral	Laminectomy	no	+	+	+	< 5%	N/A	+	No	No
4 (61y)	Differentiated	Thoracic	Dorsal instrumentation	no	+	+	+	High	N/A	+	No	No
5 (30y)	Conventional	Thoracic	Laminectomy	no	+	+	+	N/A	< 12%	+	No	Yes
6 (80y)	Conventional	Cervical	Laminectomy	no	+	+	N/A	N/A	2%	+	Yes	Yes
7 (51y)	Conventional	Cervical	Lamincetomy	yes	+	+	N/A	N/A	3%	+	No	No
8 (52y)	Conventional	Cervical	Laminectomy	no	+	+	N/A	N/A	1%	+	No	No
9 (56y)	Conventional	Lumbo-sacral	instrumentation	no	+	+	N/A	N/A	1%	+	No	Yes
10 (52y)	Conventional	Cervical	Laminectomy	yes	-	+	N/A	N/A	< 10%	+	No	Yes
11 (24y)	Dedifferentiated	Cervical	Lamincetomy	yes	+	+	N/A	N/A	40%	+	Yes	Yes
12 (56y)	Dedifferentiated	Sacral	Laminectomy	no	+	+	N/A	N/A	15%	N/A	Yes	Yes
13 (67y)	Dedifferentiated	Sacral	Laminectomy	yes	-	+	N/A	N/A	5%	+	Yes	Yes
14 (75y)	Dedifferentiated	Cervical	Instrumentation	yes	+	+	N/A	N/A	5%	+	Yes	Yes

## Complications

Regarding medical complications, one patient developed pneumonia, while another experienced hemiparesis resulting from cerebellar and pons infarction, ultimately leading to a fatal outcome during hospitalization. In particular, an 80-year-old female patient with a pre-existing ASA classification of III underwent a laminectomy and dorsal instrumentation from C0-C5 for spinal chordoma. Despite a successful surgical intervention, she developed left-sided hemiparesis (3/5 on the muscle strength scale) postoperatively. Subsequent CT imaging indicated an ischemic stroke as the underlying cause. This complication, while rare in spinal chordoma cases, highlights the elevated stroke risk associated with major surgery in elderly patients with significant comorbidities. The occurrence suggests a need for heightened perioperative monitoring and possibly more aggressive antithrombotic strategies in similar high-risk patients.

Of note, six patients underwent further surgery due to a necessary resection or renewed tumor progression after the initial operation. 9 of the 14 patients (64.3%) showed a clear remission of the preoperative neurological deficits. Five patients (35.7%) showed persistent postoperative deficits, such as tingling paresthesia with partially persistent gait ataxia. Table 3 presents detailed data on postoperative complications.

### Illustrative cases

#### Illustrative case 1

In this illustrative case, we present the management of a 76-year-old male with ASA 3, who exhibited progressive gait disturbances. An initial MRI revealed a tumorous mass extending from C1 to C4, significantly compressing the myelon. Given the patient's advanced age and severe imaging findings, we opted for a dorsal decompression of the cervical spine as the initial surgical intervention. This decision was guided by the goal of relieving myelopathy while minimizing the procedural risks associated with more extensive surgeries. Unfortunately, postoperatively, the patient experienced an aggravation of his initial gait disturbance, necessitating further diagnostic evaluation. A subsequent MRI showed a significant residual tumor mass. Based on these findings and in consultation with an interdisciplinary team, it was decided to undertake a second surgery to remove the residual tumor and provide dorsal stabilization with massa lateralis screws from C1 to C4. This approach was intended to preserve spinal stability and address the patient's deteriorating condition. Following the second surgery, the patient showed marked clinical improvement. Histopathological analysis confirmed the presence of a spinal chordoma, prompting the initiation of adjuvant radiation therapy to reduce the risk of recurrence. This case exemplifies the challenges and considerations in managing elderly patients with spinal chordomas, where initial conservative measures are closely monitored and more aggressive interventions are tailored based on evolving clinical indications and patient-specific factors. Our approach emphasizes the importance of flexibility in surgical planning and the need for ongoing assessment to optimize patient outcomes.

#### Illustrative 2 case

In this case, a 52-year-old male presented with paraparesis of both legs. Diagnostic imaging via MRI identified a large tumor mass extending from C2 to C7. Given the urgency imposed by the patient’s neurological deficits, emergency surgery was undertaken, consisting of laminectomy and tumor mass removal. Postoperatively, while the patient showed some improvement, full mobilization was not achieved. Follow-up MRI revealed residual tumor tissue, prompting a revision surgery that included additional instrumentation from C2 to C7 using massa lateralis screws to ensure spinal stability. Subsequent to this secondary intervention, the patient demonstrated significant recovery and was discharged home. Histopathological examination confirmed the diagnosis of spinal chordoma. In response to the histological findings, and following discussions at an interdisciplinary tumor conference, adjuvant radiation therapy was administered to minimize the risk of recurrence. This case underscores the critical nature of timely surgical intervention in spinal chordoma management and highlights the role of comprehensive follow-up and adaptive treatment strategies in improving patient outcomes.

Table 6 presents a comprehensive overview of the clinical progression for the six patients who required revision surgery.

**Table 6:** Detailed Analysis of Revision Surgery Reasons for Spinal Chordoma Cases

Age on admission in years	Spinal Level	Neurological Status before surgery	Surgical procedure	Reason for revision surgery	Surgical procedure
76	C1-C4	Progressive gait disturbance, ambulatory impairment	Laminectomy and decompression, C1-C4	Inadequate mobility improvement; MRI shows residual tumor	Subsequent tumor excision and dorsal instrumentation with massa lateralis screws, C1-C4
51	C2-C7	Paraparesis (KG 2–3/5)	Decompression via laminectomy from C2 to C7	Persistent pain, follow-up MRI confirms residual tumor	Further decompression and dorsal stabilization with massa lateralis screws, C2-C7
52	C1-C2	Tetraparesis (KG 2/5)	Decompression via laminectomy from C1 to C2	Aggravation of initial symptoms, follow-up MRI shows significant residual tumor	Complete tumor removal and dorsal stabilization with massa lateralis screws, C1-C2
24	C3-C4	Sensory deficits in the right arm and hand	Decompression via laminectomy from C3 to C4	Onset of sensory deficits in legs, MRI required	360-degree surgery involving corpectomy at C3 and C4 with a ventral plate from C2 to C5, and dorsal instrumentation using massa lateralis screws from C2 to C6
67	S1	Sciatica on the right side and sensory deficits on the left leg	Laminectomy at S1	Persistence of symptoms, follow-up MRI indicates residual tumor	Dorsal decompression of S1 nerve root, tumor removal with massa lateralis screws
75	C3	Sensory deficits on the left side	Dorsal instrumentation with pedicle screws from C2 to C6 and tumor removal after coiling of the left A. vertebralis	Follow-up MRI shows residual tumor	Tumor mass removal and additional dorsal stabilization with massa lateralis screws from C2 to C6

*KG* Kendall grading system for evaluation of muscle strength

In this context, Table 7 provides an overview of the most relevant literature on the surgical management of spinal chordomas, highlighting key studies and their contributions to the field.

**Table 7:** Overview of Surgical Approaches and Clinical Outcomes in Spinal Chordoma Management

Study	Localization	Surgical Approach	Surgical Margins	Adjuvant Therapy	Clinical Outcomes	Recurrence Rate
Cheng et al., 1999[5]	Sacral (19), Lumbar (4)	Varies	Wide (7), Microscopic disease remaining (5), Macroscopic disease remaining (11)	Radiotherapy (13 patients)	10-year CDFS: 59.3% for wide margins, 42% with macroscopic disease	Not reported
Baratti et al., 2003 [1]	Sacral (28)	Posterior approach (26)	Wide (11), Marginal (13), Intralesional (4)	Radiotherapy (10)	Disease-free survival: 60.6% at 5 years, 24.2% at 10 years; Overall survival: 87.8% at 5 years, 48.9% at 10 years	18% (5 cases)
Bergh et al., 2000 [2]	Sacrococcygeal (30), Cervical (5), Thoracic (1), Lumbar (3)	Varies	Wide (23), Marginal/Intralesional (16)	Radiotherapy (5 cases)	23 patients disease-free at last follow-up (mean follow-up: 8 years)	44% (17 cases)
Boriani et al., 2006[3]	Cervical (15), Thoracic (7), Lumbar (30)	Varies from intralesional to en bloc	Intralesional (20), En bloc with varying margins (18), Palliative (10)	Radiotherapy (varied based on surgical approach)	Disease-free status achieved in varied groups, with longer disease-free intervals noted in en bloc with adequate margins	66%, detailed by group
Fuchs et al., 2005 [7]	Sacral (52)	Posterior (22), Combined anterior–posterior (30)	Wide (21), Marginal/Intralesional (31)	Radiotherapy (22)	Overall survival: 74% at 5 years, 52% at 10 years	44% (23 cases)
Hsieh et al., 2011 [8]	Cervical	Combined transmandibular or submandibular and posterior approach (3), Anterior approach (2)	Wide (3), Marginal (2)	Not specified	Mean disease-free survival: 84.2 months (mean follow-up: 54.7 months)	40% (2 cases)
Meng et al., 2014[9]	Mobile Spine (58), Sacral (95)	Anterior (6), Posterior (112)	Subtotal (15), Total piecemeal (80)	Radiotherapy (14), Cisplatin/Methotrexate (107)	Local relapse-free survival: 66.7%; Overall survival: 50.1%	33% (51 cases)

## Discussion

Chordomas of the spine, although rare, present considerable therapeutic challenges. Surgical intervention plays a pivotal role in their management, often in conjunction with a multimodal treatment approach. The main goals of surgical intervention are pain relief, preservation or improvement of neurological function and effective local tumor control [7, 20]

This study highlights the critical need for a tailored therapeutic strategy that aligns surgical interventions with individual patient prognoses and neurological statuses to optimize treatment outcomes. Interestingly, all but one patient (92.9%) presented with neurological deficits and 5 patients (35.7%) had severe neurological deterioration. Our findings suggest that surgery may be the primary treatment tool, as all patients demonstrated improvement postoperatively, regardless of the specific surgical procedure employed. The surgical procedure should be determined following a thorough interdisciplinary discussion focused on the prognosis, with the primary goal of preserving neurological function. Of note, patients undergoing laminectomy only, needed further surgery due to diseases progression in 4 out of 9 cases (44.4%) compared to 2 out of 5 patients (40.0%) after cross total resection. Concerning complications, no significant differences between the groups was observed. Only one patient died due to demarking infarction of the pons and cerebellum mainly attributable to the poor baseline history.

Clinical status, as assessed by KPI and FG, has been shown by numerous studies to be one of the strongest prognostic indicators of survival in malignant tumors. The impact on prognosis in cases of spinal chordoma was extensively investigated in a study by Meng et. al. postulating preoperative KPI scores of ≥ 80% as a major prognostic factor for overall survival (OS) in chordoma patients [14]. Similarly, Frankel scores of A and B have been shown by multiple studies to be a positive prognostic factor on survival and recurrence of disease. In line with these findings, our data showed a notable shift in the distribution of Frankel grades among patients after surgery. Postoperatively, none was classified as grade A, but there was an increase in patients classified as grade B (4 patients, 28.6%), while the proportion of patients in grade D remained high (6 patients, 42.9%). Of note, the number of patients graded as grade E also increased to 4 (28.6%). The post-operative analysis of neurological and functional recovery emphasizes the crucial role of surgery in the treatment of spinal chordomas. The improvement of patients postoperatively indicates the direct impact of surgery on patient quality of life and survival. In this context, it is crucial to rigorously evaluate and discuss each patient's specific surgical requirements, taking into account their overall prognosis and eligibility for complex surgeries that may carry significant risks, including high mortality rates. Such assessments are vital to ensure that the proposed surgical interventions align precisely with the patient's health status and the potential benefits outweigh the risks involved.

Achieving clean surgical margins is paramount for optimal local control and overall survival in spinal chordoma cases [5, 15]. The surgical management of the mobile spine poses significant challenges, often requiring decisions about sacrificing nerve roots, which, although may lead to postoperative deficits, are necessary for better survival outcomes. The overarching goal is to achieve an R0 resection, which has been demonstrated to effectively control the disease. Specifically, for the cervical and thoracolumbar regions, en bloc spondylectomy remains the method of choice to ensure complete tumor resection as extensively described by Tomita et al. [21, 22]. This approach involves precise transection of pedicles following the resection of posterior elements. When the tumor is confined entirely within the vertebral body, the specimen can be removed posteriorly by rotating around the spinal cord. For extensive lesions, particularly those with significant prevertebral components or spanning multiple segments, a two-stage procedure may be necessary, wherein specimen removal is achieved via an anterior approach [9]. Post-resection, the reconstruction and stabilization of the spine are essential. Typically, this involves the use of a titanium mesh cage packed with structural fibular allograft to promote fusion, supported by pedicle screw instrumentation that extends at least two levels above and below the resected segments to secure spinal integrity [9]. Addressing the minimally invasive approaches, recent innovations have significantly enhanced surgical outcomes and reduced associated risks. Techniques like pre-operative transcatheter arterial embolization (TAE) have been shown to decrease intraoperative blood loss and simplify surgical procedures by reducing the necessity for extensive anterior approaches [25]. Additionally, the use of abdominal aorta occlusion during the resection of extensive sacrococcygeal chordomas has been noted to reduce blood loss and decrease the risk of metastasis [24]. Furthermore, the mini-open approach, which blends percutaneous pedicle screw insertion with traditional approaches for tumor excision and decompression, has proven to reduce blood loss, postoperative pain, and shorten hospital stays, highlighting its efficacy over conventional open surgeries (Barzilai et al., 2020).

Our findings are consistent with those reported in the referenced studies. According to our data, nine patients underwent decompression surgery due to the generally poor prognosis of the disease. The primary objective was to preserve and enhance the patients' neurological status, rather than achieving R0 resection. The other patients underwent a ventrolateral approach, due to their potentially favourable prognosis and there was no evidence of further metastases. All of this underscores the necessity of approaching these patients with tailored therapeutic strategies. As also documented in the study by Boriani et al., our patients undergoing en bloc resection did not experience short-term tumor recurrence, and notably, no complications associated with the surgical method were observed. Given these observations, it can be argued that while marginal resection should be a target in such diseases, the oncological status must be carefully evaluated before selecting a surgical approach. This personalized approach enables us to pursue both radical and palliative goals, emphasizing the significance of a precise surgical strategy that extends beyond mere tumor removal to optimize neurological and functional outcomes.

While the current gold standard in the treatment of spinal chordomas remains en bloc resection, it is essential to consider the potential difficulties and complications associated with these surgical procedures. In their systematic review, Denaro et al. present potential difficulties and complications associated with en bloc resection of chordomas [9]. In cervical chordomas, the primary problem is the proximity to vital organs such as the vertebral artery and nerve roots [3, 12]. In sacral chordomas, en bloc excision has been associated with potential lesions of the nerve roots. Bladder and rectal dysfunction were the most relevant surgical morbidities. There is a direct correlation between the number of preserved nerve roots and neurological functions: When both S2 roots are removed, functions are irreversibly lost; when only one S2 root is preserved, urinary and rectal functions can occasionally be restored ^17^. Fatigue fractures are another surgical complication. Bergh et al. ^19^ reported 6 fractures of the residual sacrum in a total of 18 patients who underwent high sacral resection [4]. Baratti et al. additionally found wound-related complications in 53.5% of patients with sacral chordomas [1]. In contrast to the risks and complications described in the literature, such as in the systematic review by Denaro et al., our surgical approaches proved to be relatively safe [9]. Therefore, it is essential to have a comprehensive discussion with patients and their relatives to underscore the potential risks associated with surgery, which is inevitable since it aims to halt further tumor progression and prolong overall survival rates.

Despite the apparent safety and lower immediate risk of our surgical approaches, long-term control of the disease, particularly the prevention of recurrence, remains a key challenge. The incidence of recurrence is considered as the important prognostic factor for patients with surgically treated spinal chordoma. Recurrence varies considerably after about 5 years, from 25 to 60% for cervical chordomas and from 18 to 89% for sacrococcygeal chordomas. These data are strongly affected by surgical resection margins and the use of adjuvant therapies. The main predictors of local recurrence are surgical margins and a history of previous intralesional surgery [4, 9, 11]. Another study from Meng et. also confirmed the high postoperative recurrence rate in chordomas with recurrent postoperative local tumor progression in 33.3% of cases [14]. The treatment of recurrent tumors is difficult and controversial. Contamination caused by previous surgery and altered local anatomical structures can increase the risk of recurrence. However, there are conflicting data on the role of this method in reducing the local recurrence rate. While some studies report recurrence rates of 5% to 17% after wide resection, intralesional or marginal resections show recurrence rates of 71% to 81%. In a study of 34 patients with recurrent sacral chordoma, Yang et al. were able to show the effectiveness of a repeat operation with a five-year recurrence free survival rate of 63.1% [25]. In accordance with the literature our study shows relatively high rates for local recurrence, requiring revision in 42.6% of cases, emphasizing the need for a comprehensive recurrence prevention strategy. To minimize the risk of disease recurrence, careful consideration was given not only to surgical precision and the choice of surgical techniques, but also to the potential use of adjuvant therapies to improve rates of local control and ensure long-term patient survival.

The role of adjuvant therapies, particularly radiotherapy and chemotherapy, in the treatment of chordoma poses questions due to their traditionally low response rate. Nevertheless, studies show that adjuvant radiotherapy has the potential to improve disease-free survival and local recurrence-free survival, particularly in patients whose tumors have been inadequately resected [2, 23]. In our study, eight of our 14 patients therefore received radiotherapy to minimize the risk of local recurrence and prolong disease-free survival. In addition, one patient from our cohort received systemic chemotherapy, emphasizing the limited but potentially valuable role of systemic therapy in certain cases. These multidisciplinary approaches highlight the need for a comprehensive treatment strategy to achieve the best possible outcomes for chordoma patients.

### Limitations

This study on spinal chordomas has several limitations that need to be considered when interpreting the results. The retrospective nature limits the ability to draw causal conclusions, while the small sample size of only 14 patients limits the generalizability of the results. The lack of a control group prevents a direct comparison of surgical effectiveness versus other treatment approaches. The relatively short follow-up period may also not be sufficient to fully capture long-term effects and recurrence rates. To overcome these limitations, prospective studies with larger and more diverse patient populations are needed in the future. Longer follow-up and the inclusion of control groups receiving alternative treatments would provide more robust data and allow a more informed evaluation of surgical approaches. In our study, we observed that the initial decision to opt for decompression was primarily to preserve neurological function, which is often compromised in acute presentations. This choice was guided by the need to balance immediate clinical needs with long-term treatment outcomes. Following initial decompression, cases were reassessed at an interdisciplinary conference where decisions regarding further ventrodorsal stabilization and adjuvant treatment approaches were made, taking into consideration the patient’s overall health status, age, comorbidities, and the associated surgical risks. In addition, data collection across multiple centers would be desirable to optimize clinical practice and improve treatment outcomes for patients with spinal chordomas.

### Conclusions

In conclusion, surgical resection of spinal chordomas is a safe and effective treatment option that has the potential to improve neurological function. Given the scarcity and complexity of spinal chordomas, it is imperative to accurately determine patient prognosis, identify specific needs, and assess eligibility for extensive surgery within an interdisciplinary framework at a specialized center. Only under these conditions can we ensure that each patient receives the most effective and personalized treatment plan. This approach is essential for optimizing surgical outcomes and improving the quality of life for those affected by this challenging disease.

## Data Availability

The datasets generated during and/or analyzed during the current study are available from the corresponding author on reasonable request.

## Code availability

The code supporting the findings of this study is available from the corresponding author upon reasonable request.
